# Supplementary material for: Surveillance Program of Clinical Samples for Polio and Non-Polio Enteroviruses in the Slovak Republic during the 1958–2020 Period
Source: Viruses. 2022 Sep 3;14(9):1957. doi: 10.3390/v14091957 (PMC9506458; doi:10.3390/v14091957)
Supplement: Supplementary file 1 [file viruses-14-01957-s001.zip › Supplementary table S1.pdf]

Supplementary Table S1

Polio vaccination program in Slovakia for the period 1957-2020

Vaccination	**Years**	**Vaccination against polio**
Basic vaccination	1957-1959	IPV*: Salk inactivated vaccine
	1960-2004	OPV**: Sabin live attenuated vaccination campaigns (March-May), children after 10 weeks of life up to 1 year
	2005 – till today	IPV: Salk inactivated vaccination 3 doses, at 3, 5, and 11. months of age
First Booster dose	1960-2005	OPV: Sabin live attenuated vaccine campaigns continue from March to May
	2006 - till today	IPV: Salk inactivated vaccine 1 dose at 6 years of age
Second Booster dose	1960-1984	OPV: Sabin live attenuated vaccination, 1 dose in the second grade of school that is at age 7-8 years.
	1985-2006	OPV: Sabin inactivated vaccine, in the 7th grade that is age 12-13 years
	2007-till today	IPV: Salk inactivated vaccine 1 dose at the age of 13

Note = Poliomyelitis vaccination was and is part of the compulsory vaccination of children in the Slovak Republic

* IPV = Inactivated vaccine

** OPV = Oral live attenuated vaccine
