# Supplementary material for: Surveillance Program of Clinical Samples for Polio and Non-Polio Enteroviruses in the Slovak Republic during the 1958–2020 Period
Source: Viruses. 2022 Sep 3;14(9):1957. doi: 10.3390/v14091957 (PMC9506458; doi:10.3390/v14091957)
Supplement: Supplementary file 1 [file viruses-14-01957-s001.zip › Supplementary table S2.pdf]

Supplementary Table S2

Isolations on cell cultures of EVs from clinical samples in the Slovak Republic from 1958 to date

**Period**	**Cell cultures**	**Animals**	**Methods**	**Material**
**1958- 1990**	1958- buffalo green monkey kidney cells (BGM)	suckling mice aged 12-36 hrs	1958 - WHO Geneva Comission experts [8]	Mainly stool samples (at least 2 samples between 24-48 hrs), but also cerebrospinal fluid, throat, nasal and conjunctival swabs, autopsy materials<br><br>2 to 5 cell culture flasks for 1 sample, on at least 2 types of cell cultures
	1962 – HeLa (epithelial cervical cancer cells, human amniotic cells (primary cultures from placenta) and human embryonic cells			
	1968 - human embryonic lung cells (LEP19)			
	1984 - Vero (African green monkey kidney) cells (for VNT)			
**1991-1996**	cell lines human rhabdomyosarcoma-derived (RD) and human epidermoid carcinoma (Hep2)		1993 - WHO Manual for the virological investigation of poliomyelitis [6]	
**1997-present day**	cell lines human rhabdomyosarcoma-derived (RD), human epidermoid carcinoma (Hep2) and genetically modified mouse cell lines with the human poliovirus receptor (L20B)		2004 - Polio laboratory manual, 4th ed. World Health Organization. [26]	
			2015 - Enterovirus surveillance guidelines. [10]	
			2017 - S1. Supplement to the WHO Polio Laboratory Manual An alternative test algorithm for poliovirus isolation and characterization [28]	

Note – the limitation, that these methods were probably not implemented in all laboratories at the same time, so their use was overlapping at the changing of methods, that the time data may not be completely accurate, the same is true for cell cultures.
